# Gender Diversity and Work–Life Conflict in Changing Times

**DOI:** 10.3390/ijerph17239009

**Published:** 2020-12-03

**Authors:** Luo Lu, Shu-Fang Kao, Ting-Ting Chang, Cary L. Cooper

**Affiliations:** 1 Department of Business Administration, National Taiwan University, Taipei City 10617, Taiwan; 2 Department of Applied Psychology, Hsuan Chuang University, Hsinchu City 300, Taiwan; d89227002@gmail.com; 3 Department of Industrial Management, Lunghwa University of Science and Technology, Taoyuan City 333, Taiwan; tinapc@ms24.hinet.net; 4 Alliance Manchester Business School, University of Manchester, Manchester M13 9PL, UK

**Keywords:** biological sex, gender diversity, masculinity traits, femininity traits, work and family demands, work and family conflict, Chinese culture

## Abstract

The aim of the study is to contribute to the “well-being, diversity, equity, and inclusion” dialogue of the post-pandemic era. Specifically, we explored the joint effects of biological sex and gender diversity in self-identity on the role demands—work and family conflict relationships. To advance the inclusion of scientific knowledge, the present study was conducted in the cultural context of a Chinese society. We surveyed a sample of 317 Taiwanese employees. We used structured questionnaires to collect data on biological sex, gender identity (self-endorsement on masculinity and femininity traits), work and family demands, work-to-family conflict (WFC), and family-to-work conflict (FWC). We found two sets of significant three-way interactions (sex × femininity × role demands) in predicting work and family conflict. First, for men, identifying with high femininity traits strengthened the positive relationship between work demands and FWC; for women, identifying with low femininity traits strengthened the same relationship. Second, for men, identifying with high femininity traits strengthened the relationship between family demands and WFC; for women, identifying with low femininity traits strengthened the same relationship. Our findings highlight the importance of jointly examining the biological, psychological, and social aspects of gender on the work and family interface. Contextualizing in an Eastern cultural tradition, we put the spotlight on societal pressure on people of nontraditional gender identities.

## 1. Gender Diversity and Work-life Conflict in Changing Times

In 2020, the world has been engulfed in a triple pandemic, e.g., COVID-19, the economic recession, and the social revolution. In the post-pandemic era, the creation, communication, and application of scientific knowledge on employee well-being, equity, diversity, and inclusion deserve more concerted efforts from academia of all disciplines. Additionally, in the likely prolonged recession, intensifying work demands will expose employees to greater risk of work and family, which is defined as occurring when participating in one role is made more difficult by virtue of participating in the other role [1]. The work–family conflict is bidirectional, as work may interfere with family (work-to-family conflict, WFC) and family may interfere with work (family-to-work conflict, FWC). Responding to the challenge, the present study aimed to highlight the plethora of gender diversity manifested as self-identified gender traits and its linkage to well-being through work and family role enacting. Further, contributing to the inclusiveness of scientific content, our study targeted the under-represented Asian populations in the mainstream work and family literature.

The increasing labor participation of women worldwide has been one impetus for the championing of gender diversity at work. The linkage between individuals’ sex and the work and family interface is thus a familiar question for work and family scholars. However, systematic reviews revealed that findings of sex differences are very inconsistent. Eby et al. [2] concluded in their meta-analysis that evidence is mixed whether men and women reported different levels of WFC and FWC. For instance, some researchers found *no* sex difference in WFC [3], others found that men reported higher WFC than women [4], or women reported higher WFC than men [5]. A later review found that sex had no direct effect on either WFC or FWC, and rather inconsistent moderating effect on the conflict–satisfaction relationships [6]. To understand the inconsistency of findings, researchers have called for the reconfiguration of “sex” as a multifacet construct encompassing biological, psychological, and sociological aspects of role prescriptions [6,7]. We set out to examine the interactive effects of biological sex, psychological gender (gender traits or gender role orientation), and role demands on WFC/FWC. Our study contributes to the literature by suggesting that it is the intersection of biological sex and psychological gender diversity (masculinity and femininity traits) that makes a difference in the individuals’ perceptions of role demands and their experiences of work and family conflict. We thus not only add to the scarce empirical evidence of within-sex differences of men and women in role practicing, our reconfiguration of “sex” also contributes to the post-pandemic gender diversity agenda.

The experience of work and family conflict depends on both the individual’s role enacting and the contextual factors. Our second contribution is to extend the generalization of the gender diversity construct to an Eastern society to advance inclusive and equitable contents of scientific knowledge. The cultural context is poignant as “gender” refers to the sociocultural, psychological, and behavioral attributes associated with one’s biological sex as decreed normative in a particular society [8]. In Taiwan, where our study was conducted, traditional Chinese values on gender role expectations still prevail, as shown in a nationwide survey that most people (40.7%) endorsed the traditional attitude of “men as breadwinners, women as homemakers” [9]. We thus explored the three-way interaction of biological sex, psychological gender traits, and role demands on WFC/FWC to expand what we already know in the more egalitarian Western societies to the culturally different Eastern societies. We add to the emerging studies in Eastern cultural contexts (e.g., Sri Lanka: [10]; China: [11]) to correct the over-reliance of work and family research on samples from Western cultures (see [12] for a review) and to advance the inclusion of knowledge.

## 2. Theoretical Framework and Hypotheses Development

### 2.1. Sex Differences and Gender Diversity in the Work and Family Interface

In line with the theoretical reconfiguration in gender research [13,14,15], we distinguish the term sex as the binary biological category of male and female, from gender as the psychosocial implications of being male or female. This terminology (sex vis-à-vis gender) is hereafter used in this paper to elucidate how men and women enact the work and family roles. 

Our theoretical distinction however, departs from the tradition of work and family literature, where gender has often been operationally equalized to the biological sex, committing the “biopsychological equivalence fallacy” [7]. In other words, work and family researchers often assume that biological men would identify and comply with the socially prescribed male role of “breadwinners,” whereas biological women with the female role of “homemakers.” The operational equivalence of sex with gender masks the within-sex variations in role-enacting related to gender diversity such as varied gender self-identities. Korabik et al. [7] thus urged researchers to explore the intrapsychic aspects of gender that influence not only individuals’ identities but also their behaviors, the roles they choose to enact, and how they choose to enact them. One such psychological gender construct is the gender role orientation theory [16].

Gender role orientation (hereafter GRO) posits that individuals’ self-concept inherently contains personal characteristics, which in turn influence the gender-typing of their behavior and cognitions [16]. The two independent dimensions of masculinity and femininity traits capture the cultural descriptions for the identities of men and women. Masculinity describes the beliefs about the degree to which one holds traits that are associated with men (aggressiveness, competitiveness, and dominance), and femininity describes the beliefs about the degree to which one holds traits that are associated with women (sensitiveness, warmth, and compassion) [16,17]. Livingstone and Judge [18] postulate that GRO represents an individual’s attitudinal recognition with gender roles, such that men are more likely to exhibit “masculine” traits that are viewed as instrumental in the work domain (e.g., aggressiveness, decisiveness, independence), while women are more likely to exhibit “feminine” traits that are conducive to family life (e.g., compassion, nurturance, sensitivity to the needs of others). Gender diversity as self-identities of psychological traits thus sets expectations for enacting the individual’s role [19]. 

Gender role theory postulates that society sets norms for what role behaviors are appropriate and the roles that men and women should emphasize: women’s proper place is in the home and men’s in the workplace [20]. Research on the GRO theory has shown that masculinity traits were related to avowed work role importance, whereas femininity traits to avowed family role importance [21]. This is the work and family role enacting for traditional men and women as decreed in a traditional society. Through the process of self-identification, the traditionals (i.e., masculine men, feminine women) will regard their work or family role as more important than the other role. However, Eagly and Diekman [22] pointed out that gender roles are malleable to some extent by men and women, especially during profound social changes in gender attitudes. When societies move towards greater gender equality, there is increasing tolerance for gender diversity such as within-sex variations in the identification of gender traits. Recent evidence showed that cultural change may affect individual personalities as women increased endorsement of masculine traits while men maintained their nonendorsement of feminine traits [23]. 

Studies have shown that within-sex variation is as critical as those between the sexes to explain differences among individuals’ experiences of work and family conflict [24,25,26]. Thus, we propose that traditional vis-a-vis nontraditional men and women may have different perceptions of work and family roles, and different experiences of work and family conflict. Because the psychological meaning differs immensely for men/women identifying with female/male appropriate traits, we expect there to be a three-way interaction between the focal person’s sex, his/her identified gender traits, and work and family demands in predicting WFC/FWC. In a low gender egalitarian context like the Chinese society, traditional men draw their main identity from their bread-earner role, whereby their job-related demands take precedence over family responsibilities, and the family domain is more permeable than vice versa; on the other hand, traditional women draw their main identity from their homemaker role, whereby their family-related responsibilities take precedence over work demands. In the present-day Taiwan, women have been given increasing opportunities for education and career, while men have been increasingly encouraged to participate in family affairs. Nevertheless, research in Taiwan [9,27] shows that for most women it is still “family first,” for which they are willing to make compromises/adjustments in their careers, for example, rejecting promotions and/or relocation opportunities with good career prospects, which may take them away from their families. On the other hand, for most men, it is still “career first,” for which they are willing to be excessively available for work, for example, working “always on” and seeking expatriation opportunities with advancement prospects. One study in India [28] found that women “traditionals” (nonegalitarians) experienced more FWC than men “traditionals,” suggesting across-sex differences among traditionals. However, it is unknown whether within nontraditionals (e.g., feminine men, masculine women) if there are also across-sex differences. 

### 2.2. Gendered Traits and the Prominence of Work and Family Role Demands

Following traditional gender role values, men should emphasize more on the work role, and take on the duty of bread winners; while women should invest more energy on the family role, and act as caregivers [20]. Thus, when competing role demands happen, traditional men and women will respond differently in their resource investment according to their respectively identified gender roles. Specifically, for traditional men (those who identify with masculinity traits), when work demands increase, they will mobilize their resources to fulfill the primary role obligation, and may spend less time and energy in family life with null or low feelings of conflict [11,29]. Similarly, for traditional women (those who identify with femininity traits), when family demands increase, they will mobilize their resources to fulfill the primary role obligation, and may spend less time and energy in work life with null or low feelings of conflict [27,30]. In other words, masculine men and feminine women will prioritize work or family role at the expense of the other role. This is a regulatory resource investment behavior to enact the role identifications. 

However, the demarcation between work and family is blurred for nontraditional men and women as they do not identify with traditional gender traits. For men who identify with nontraditional feminine traits, compared to their traditional counterparts who draw self-identity primarily on the work role, femininity traits make them more sensitive to family-related responsibilities [21]. Powell and Greenhaus [31] found a positive association between femininity traits and the importance of family role for both men and women. When work requires more energy exertion, low masculine or high feminine men will be more sensitive to the intrusion of family life detracting from their work performance. As prioritizing the family role is not socially desirable for men, they will experience more intense FWC. For women who identify with high masculine or low feminine traits, compared to their traditional counterparts who draw self-identity primarily on the family role, masculinity traits make them more inclined to pursue career achievements [21]. Research has found that women with the nontraditional gender role identity had higher achievement motivation and pursued career success more rigorously [32,33]. Being more committed to the work role, though not socially desirable for women, high masculine or low feminine women will be more perceptive to the competing expectations in the family realm, especially when they wish to devote themselves to meet work challenges. In sum, when nontraditional men and women face increasing work demands, they may not opt for sacrificing family or work life, as this is inconsistent with their self-identities [34]. While trying harder to protect the roles that are akin to their self-identities, albeit incongruent with society expectations of their sexes, the feelings of distress and conflict will be heightened [35]. Simultaneously examining sex and gender traits, we focused on *within-sex variations* to explain how gender diversity influence the roles men and women choose to enact and how they choose to enact them. We thus hypothesized:

**Hypothesis** **1** **(H1).** *There will be a three-way interaction between sex and masculinity in the relationship between work demands and FWC such that masculinity will moderate the relationship between work demands and FWC. Specifically, (a) the positive relationship between work demands and FWC will be weaker for men with higher rather than lower masculinity (for men: traditionals < nontraditionals); (b) the positive relationship between work demands and FWC will be stronger for women with higher rather than lower masculinity (for women: nontraditionals > traditionals).*

**Hypothesis** **2** **(H2).** *There will be a three-way interaction between sex and femininity in the relationship between work demands and FWC such that femininity will moderate the relationship between work demands and FWC. Specifically, (a) the positive relationship between work demands and FWC will be stronger for men with higher rather than lower femininity (for men: nontraditionals > traditionals); (b) the positive relationship between work demands and FWC will be weaker for women with higher rather than lower femininity (for women: traditionals < nontraditionals).*

Similarly, when family demands increase, we expect that nontraditional men and women will experience increased WFC. For nontraditional men, compared to their traditional counterparts who draw self-identity primarily on the work role, the femininity traits make them more inclined to pursue family happiness through involvement and participation [21]. When family duty calls, nontraditional men will be more resentful of the competing demands of the work role that depletes their energy and constrains their time. As prioritizing the family role is against the social prescription for men, they will experience more intense work-to-family conflict. For *nontraditional women*, compared to their traditional counterparts who draw self-identity primarily on the family role, and willingly accept devotion to family as the role script, the masculine traits make them more inclined to pursue career achievements and regard family responsibilities as “duties/obligations.” As it is against the social prescription for women to prioritize the work role, they will feel “forced” to fulfill the family obligations nonetheless, thus experiencing more intense work-to-family conflict. We thus hypothesized:

**Hypothesis** **3** **(H3).** *There will be a three-way interaction between sex and masculinity in the relationship between family demands and WFC such that masculinity will moderate the relationship between family demands and WFC. Specifically, (a) the positive relationship between family demands and WFC will be weaker for men with higher rather than lower masculinity (for men: traditionals < nontraditionals); (b) the positive relationship between family demands and WFC will be stronger for women with higher rather than lower masculinity (for women: nontraditionals > traditionals).*

**Hypothesis** **4** **(H4).** *There will be a three-way interaction between sex and femininity in the relationship between family demands and WFC such that femininity will moderate the relationship between family demands and WFC. Specifically, (a) the positive relationship between family demands and WFC will be stronger for men with higher rather than lower femininity (for men: nontraditionals > traditionals); (b) the positive relationship between family demands and WFC will be weaker for women with higher rather than lower femininity (for women: traditionals < nontraditionals).*

## 3. Method

### 3.1. Procedure and Participants

The participants in our study were employees working in different organizations of diverse industries across Taiwan. Some of our participants were part-time MBA students and some were recruited through contact managers in organizations. The faculty and managers ascertained the commitment for participation before sending out questionnaires using email or hard copy. A cover letter accompanying the questionnaire informed participants on the purpose of the study and assuring them of anonymity and confidentiality. Upon return of the completed questionnaire, the participant was given a small gift as a token of appreciation. Three hundred seventeen questionnaires were given out and all were returned (response rate: 100%) with usable data. Based on the mean scores of the study variables, we systematically examined differences between participants who filled out the hard-copy questionnaire versus those who completed the e-version through email. Analyses revealed no significant differences overall. We thus pooled the data for further analysis (*N* = 317).

The sample was 53.3% male and 46.7% female, with a mean age of 35.24 (SD = 11.91), and mean job tenure of 13.62 years (SD = 10.56). Most participants (54.3%) had college-level education and over a quarter of the respondents (27.5%) were managers. Just under half of the sample (45.7%) were married or cohabiting, while the rest were single, widowed, separated, or divorced (54.3%). Regarding family circumstances, 43.8% of our participants had children, and 42.4% were living with parents or in-laws.

### 3.2. Measures

Gender traits. The Chinese version “Sex Role Inventory” was revised from “Bem’s Sex Role Inventory” (BSRI) [16] with Chinese trait descriptors and validated with Chinese subjects [36]. The inventory has a 20-item masculinity (MAS hereafter) scale pertaining to instrumental-agenic traits, and a 20-item femininity (FEM hereafter) scale pertaining to expressive–communal traits. There was evidence supporting construct validity of these two scales [37]. Sample items (adjectives) of the MAS scale are “ambitious” and “independent.” Sample items (adjectives) of the FEM scale are “warm” and “empathetic.” Five-point rating scales were used (1 = not true for me, 5 = extremely true for me), with higher scores representing higher identification with the MAS and FEM traits. Following Bem’s [16] theory, masculinity and femininity traits are two discernible psychological dimensions that coexist within an individual. Thus, they were analyzed as two independent variables in the present study. The internal consistency reliability of the MAS scale was 0.92 and that of the FEM scale was 0.89. 

Work demands. Quantitative workload was used to indicate work demands. Five statements from the quantitative workload inventory (QWI) [38] describe quantitative aspects of work demands (e.g., “How often is there a great deal to be done?”). Respondents answered each statement by indicating the frequency of occurrence, from 1 (never happened) to 5 (always happening), with higher scores representing higher work demands. The internal consistency of the QWI was 0.84 in the present study.

Family demands. Family responsibility was used to indicate family demands. Three statements from the family responsibility scale (FRS) [39] describe quantitative aspects of family demands (e.g., “How often do you feel that your family makes too many demands on you?”). Respondents answered each statement by indicating the frequency of occurrence, from 1 (never happened) to 5 (always happening), with higher scores representing higher family demands. The internal consistency of the FRS was 0.87 in the present study.

WFC and FWC. The work–family conflict scale (WFCS) [40] was used to assess WFC and FWC separately. Sample items are: “The amount of time my job takes up makes it difficult to fulfill family responsibilities” (WFC), and “I have to put off doing things at work because of demands on my time at home” (FWC). Respondents rated the items on a five-point Likert scale (1 = absolutely incorrect, 5 = absolutely correct), with higher scores representing higher conflict in both directions. The internal consistency of the WFC scale was 0.92 and that of the FWC scale was 0.85 in the present study.

Demographics. Demographic information on sex (coded as men = 1, women = 0), age, marital status (coded as married = 1, not married = 0), living arrangement (coded as living with parent = 1, not living with parent = 0), tenure on the job (in years), and rank (coded as managers = 1, employees = 0) were recorded. These were used as control variables in all the subsequent analyses.

## 4. Results

### 4.1. Descriptive Analysis

Prior to the hypotheses testing, bivariable correlations were computed; the results are shown in Table 1. It is important to note that sex correlated with neither MAS nor FEM, and neither MAS nor FEM correlated with WFC or FWC. However, both work and family demands positively correlated with WFC and FWC. As can be seen in Table 1, sex (biological men) positively correlated with FWC, whereas being married positively correlated with FEM, and holding a managerial position positively correlated with MAS; thus we controlled for all demographic characteristics and job variables in the subsequent analyses.

### 4.2. Confirmatory Factor Analyses (CFA) and Testing for Common Method Variance (CMV)

To ensure that the constructs of our research model can be meaningfully distinguished, we conducted CFA to compare a series of measurement models. Specifically, our six-factor research model where items loaded on the theoretically assumed correlated latent factors (i.e., MAS, FEM, work demands, family demands, WFC, FWC) was set against alternative solutions (three-factor model that combined MAS and FEM, work and family demands, WFC and FWC: χ^2^/df = 4.16, CFI = 0.54, NFI = 0.47, RMSEA = 0.10, SRMR = 0.13; and one-factor model where all items loaded on one general latent factor: χ^2^/df = 5.47, CFI = 0.34, NFI = 0.30, RMSEA = 0.12, SRMR = 0.14. The six-factor solution consistently fitted the data better (χ^2^/df = 2.28, CFI = 0.91, NFI = 0.87, RMSEA = 0.04, SRMR = 0.05), thus supporting the structure of our research model. As self-report measures may increase the threat of common method variance (CMV) bias [41], we examined an alternative model that matched our hypothesized research model except for the inclusion of an unmeasured, latent method factor [41]. The model fit was very poor (χ^2^/df = 8.69, CFI = 0.56, NFI = 0.48, RMSEA = 0.18, SRMR = 0.18), indicating that it was unlikely that any substantial proportion of variability in responses could be attributable to the method factor.

### 4.3. Hypotheses Testing

Following Baron and Kenny’s [42] suggestion for testing and reporting moderating effects, we conducted a series of hierarchical regression analyses to test our hypotheses. Predictors were standardized and interaction terms were created from these standardized predictors. In keeping with Bem’s theoretical proposition and the established gender trait research paradigm, MAS and FEM were tested separately in regression models. When testing H1 and H2, FWC was the dependent variable. We first entered all the control variables in the regression models. At the second step, we entered workload as the independent variable. At the third step, we entered sex and gender traits (MAS or FEM) as moderators. At the fourth step, two-way interaction terms (workload × sex, workload × MAS or FEM, sex × MAS or FEM) were entered. At the final step, the three-way interaction term (workload × sex × MAS or FEM) was entered. The results are reported in Table 2 (for MAS) and Table 3 (for FEM).

Results in Table 2 showed that having controlled for effects of demographics, workload, sex, gender trait of MAS, and two-way interactions, the focal three-way interactive effect pertaining to masculinity was not significant in predicting FWC (Model 5). Thus, Hypothesis 1 was not supported. Results in Table 3 showed that having controlled for effects of demographics, workload, sex, gender trait of FEM, and two-way interactions, the focal three-way interactive effect pertaining to femininity was indeed significant in predicting FWC (Model 5). Thus, Hypothesis 2 was supported. 

**Figure 1:**
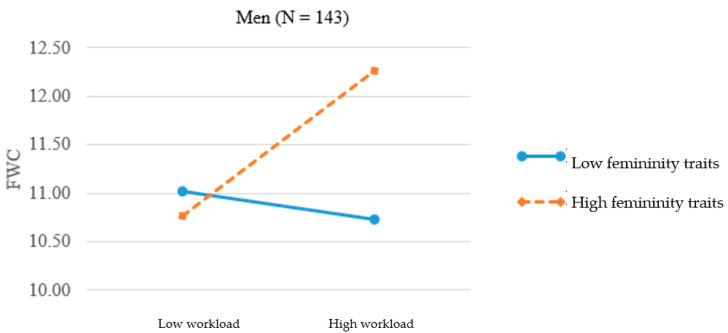
Interaction of femininity traits and workload on FWC for men.

**Figure 2:**
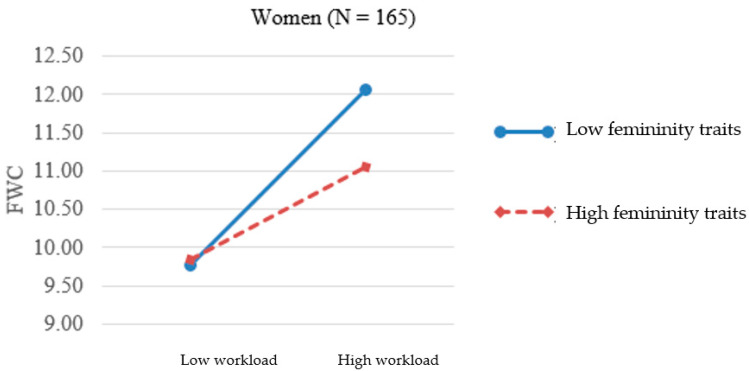
Interaction of femininity traits and workload on FWC for women.

As we argued earlier, for men and women, identifying with masculinity and femininity, depending on whether such traits are assigned to their sex or those to the opposite sex, has very different meanings and subsequent effects on their behaviors and experiences. Thus, it is meaningful to plot the significant three-way interaction effect at one standard deviation above and below the mean [43] separately for men (Figure 1) and women (Figure 2). In Figure 1, the simple slope was larger for the high feminine men (0.34, *p* < 0.001) and smaller for the low feminine men (–0.03, n.s.). The result provides support for H2a, namely, nontraditional (feminine) men experienced greater FWC when work demands went higher. In Figure 2, the two slopes for high and low feminine women were 0.13 (*p* < 0.05) and 0.30 (*p* < 0.001). The result lends support for H2b, showing that the relationship between workload and FWC was stronger for nontraditional (low feminine) women when work demands went higher. 

The same logic and procedure described above were followed when testing for H3 and H4. The only differences were this time WFC was the dependent variable and family responsibility was the independent variable. The results of these two sets of regression analysis are reported in Table 4 (for MAS) and Table 5 (for FEM). 

Results in Table 4 show that having controlled for effects of demographics, family responsibility, sex, gender trait of MAS, and two-way interactions, the focal three-way interactive effect pertaining to masculinity was not significant in predicting WFC (Model 5). Thus, Hypothesis 3 was not supported. Results in Table 5 showed that having controlled for effects of demographics, family responsibility, sex, gender trait of FEM, and two-way interactions, the focal three-way interactive effect pertaining to femininity was indeed significant in predicting WFC (Model 5). Thus, Hypothesis 4 was supported.

**Figure 3:**
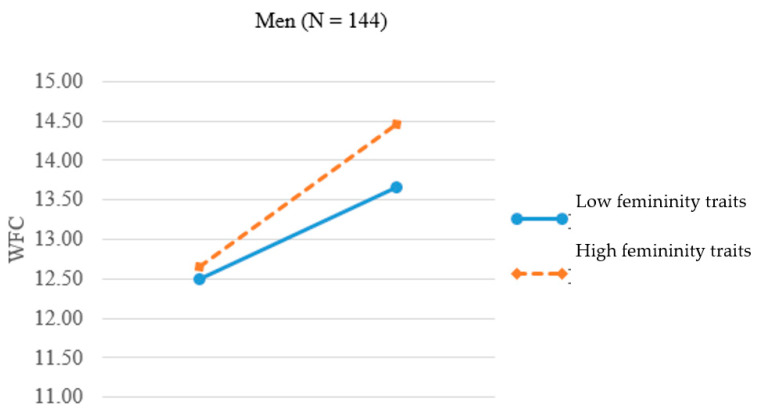
Interaction of femininity traits and family responsibility on WFC for men.

**Figure 4:**
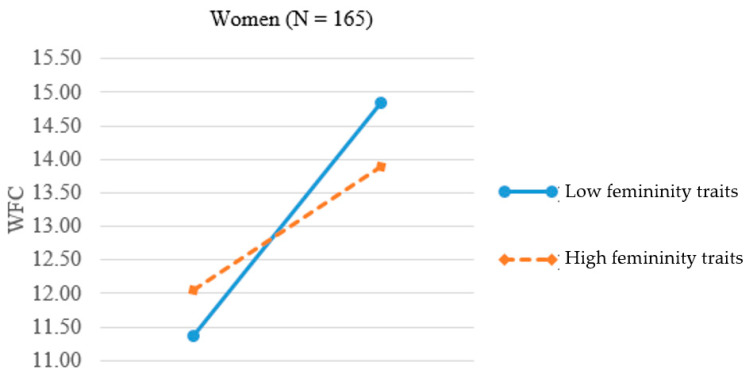
Interaction of femininity traits and family responsibility on WFC for women.

We again plotted the significant three-way interaction effect separately for men (Figure 3) and women (Figure 4). In Figure 3, the two slopes for high and low feminine men were 0.24 (*p* < 0.01) and 0.20 (*p* < 0.05). The result provides support for H4a, namely, nontraditional (feminine) men experienced *greater* WFC when family demands went higher. In Figure 4, the two slopes for high and low feminine women were 0.21 (*p* < 0.05) and 0.33 (*p* < 0.001). The result lends support for H4b, showing that the relationship between family responsibility and WFC was stronger for nontraditional (low feminine) women when family demands went higher.

## 5. Discussion

Previous studies of work and family have found either nonsignificant or inconsistent sex differences on levels of conflict between men and women (see reviews such as [2,6]). We instead viewed “sex” as a multifacet construct with biological, psychological, and social implications for individuals to enact work and family roles. The present study is the first to examine the three-way interaction effects of biological sex, gender diversity, and role demands on the bidirectional work and family conflict in a Chinese society undergoing rapid social changes. To recap, we found that both nontraditional (high feminine) men and nontraditional (low feminine) women experienced greater FWC when work demands went higher, compared to their traditional same-sex counterparts. Furthermore, the same nontraditional groups of men and women experienced greater WFC when family demands went higher, compared to their traditional same-sex counterparts. 

### 5.1. Theoretical Contribution

Our findings make theoretical contributions to the existing work and family literature on the following fronts. First, through systematic examination of gender diversity in self-identity, our study highlights the folly of “biopsychological equivalence” prevalent in the literature [7]. Our results clearly show that when gender self-identity is measured, biological sex had no relation with an individual’s endorsement of gender traits. The gender role orientation theory does not make the assumption of biopsychological equivalence; i.e., both men and women can identify with high or low masculinity and femininity traits [7,16]. To the extent that self-identity directs behavior, individuals will seek and be satisfied with roles that maximize cognitive consistency [44]. For example, to maintain a positive self, individuals would select a coping strategy that is congruent with their gender identity, thereby satisfying the need for self-consistency [45]. Our nuanced approach to include the biological, psychological, and social aspects of “sex” will advance a more equitable understanding of gender diversified men’s and women’s experiences of work and family in the post-pandemic society.

Second, using biological sex as a proxy for gender has led to overlooking the within-sex variations in work and family roles. Our simultaneous examination of biological sex with psychological gender traits unraveled the protean nature of contemporary gender role identification, such as the “family men” and “career women.” Our research demonstrates the utility of gender traits as individual-level psychological constructs and clarifies that it is the intersection of sex and gender role traits that makes a difference in individuals’ perceptions of role demands and their experiences of conflict. 

Third and most importantly, we consistently found that the identification with nontraditional gender traits (those that differ from the prescribed gender traits for one’s biological sex) exposed individuals to a vulnerable position in the work and family interface. Our results highlight the powerful macrocultural contexts that have been largely overlooked in the Western-dominated work and family research. It is important to note that in the East, “family men” and “career women” may suffer from more elevated work and family conflict, compared to their traditional counterparts (e.g., breadwinning men and homemaking women). The systemic examination of gender diversified self-identities (e.g., traditional men with high masculinity, nontraditional men with high femininity, traditional women with high femininity and nontraditional women with high masculinity) lends greater weight to advance gender equitable knowledge. It is thus worth noting that masculinity is not potent in the trilogy of sex, gender, and work and family interface, whereas femininity may hold the key for the adjustment of both men and women. More precisely, it is the feminine men and nonfeminine women who face the precarious circumstances when work and family demands mount. The commonality between the two groups are their nontraditional gender identities: men identifying with female traits and women not identifying with female traits, thus possibly detracting them from the traditional male role of work and female role of family. Although empirical findings are still scarce, the reality for “moving ahead of the tide” may be less than rosy. A recent study in mainland China found that among male managers, those with egalitarian gender attitudes suffered more negative consequences of WFC [11]. Our results complement and extend this preliminary evidence to both men and women, employees, and managers, on both directions of the work and family spillover (WFC and FWC). In a Chinese society that puts great emphasis on conformity and fitting-in, the social pressure on those nonconformists is phenomenal [46]. Such pressure is likely to result in threats to self-worth and conflict over self and others’ expectations. All of these render the nonconformists stress-prone in juggling work and family roles. Our findings are in line with the resource view [47]. In a transitional society, while nontraditional men and women desire to immerse in their self-chosen role, they are still obligated to perform the societal prescribed role, resulting in greater resource competition and depletion, such as extended working hours and reduced leisure time [35,48]. One study in Taiwan found that those who upheld stronger nontraditional values (i.e., prioritizing the individual over the collective) suffered higher WFC/FWC when role demands increased [49]. Along with encouragement of diversity and equity in the post-pandemic era, the challenges for taking the road less trodden still need to be better researched and demythologized.

### 5.2. Managerial Implications

The stronger associations between role demands and work–family conflict for nontraditional men and women suggest that organizations should invest more efforts to promote equity and inclusion, especially targeting those with nontraditional gender traits with work–life balance resources. Organizational interventions such as flexible work and supervisory support for family values have been shown to decrease work and family conflict [12,50]. Organizations should also establish policies to create a level playing field for all employees to fulfill their aspirations, regardless of sex and gender role orientations.

### 5.3. Limitations and Future Research Directions

Our study has limitations. First, our data were cross-sectional and self-reported, causal relationships between role demands and work and family conflict cannot be ascertained. Future research should adopt a longitudinal study design to justify causality. As for CMV, our tests confirmed that all research variables were empirically distinguishable and the bias due to common method variance was low. Researchers have argued that interactive effects are less affected by CMV [51]; it is still valuable to obtain other sources of data in future research, such as supervisor or spouse ratings of role demands. Third, due to various restrictions, we could only use a convenient sampling method to obtain data. Comparing to the profile of a published national probability sample of full-time workers in Taiwan [50], our sample was younger (35.24 vs. 40), had more women (46.7% vs. 34%), fewer married (45.7% vs. 70.1%), and about the same proportion of managers (27.5% vs. 29.1%). Thus, we cannot claim the representativeness of Taiwanese working population, and our results have to be interpreted with due caution and reservation. Last but not the least, as our study is contextualized in the Taiwanese conditions, results may not generalize to other cultures. Future research on cross-cultural comparisons is encouraged. 

## 6. Conclusions

Conducted in a transitional Chinese society, the present study simultaneously examined the three-way interaction effects of biological sex, gender diversity in self-identities, and work and family demands on WFC/FWC. We unraveled both the within-sex and across-sex differences on the work and family interface, emanating from individuals’ diverse gender identities. Men endorsing high femininity traits and women endorsing low femininity traits are more vulnerable compared to their traditional counterparts. The underlying mechanisms of such vulnerability highlight the pressure of nonconformance to traditional gender role scripts. In the post-pandemic changing times, researchers should thus take into account the full biopsychosocial implications of gender to achieve an equitable and inclusive understanding of men’s and women’s experiences of work and family roles.

## Figures and Tables

**Table 1:** Interrelations among research variables (*N* = 317).

	Mean	SD	1	2	3	4	5	6	7	8	9	10	11	12
1. Age	35.24	11.91	1.00											
2. Sex	0.47	0.50	−0.01	1.00										
3. Marital status	0.46	0.50	0.74 ***	0.01	1.00									
4. Number of children	0.44	0.50	0.77 ***	0.00	0.85 ***	1.00								
5. Living arrangement	0.58	0.49	−0.45 ***	0.00	−0.33 ***	−0.32 ***	1.00							
6. Rank	0.28	0.45	0.20 ***	0.09	0.17 **	0.14 *	−0.12 *	1.00						
7. WFC	13.04	3.86	0.11	0.05	0.18 **	0.13 *	−0.06	0.10	1.00					
8. FWC	10.91	3.11	0.17 **	0.12 *	0.24 ***	0.22 ***	−0.08	−0.04	0.55 ***	1.00				
9. Workload	17.83	3.51	0.07	−0.10	0.11 *	0.05	−0.12 *	0.20 ***	0.46 ***	0.18 **	1.00			
10. Family responsibility	7.46	2.62	0.30 ***	−0.06	0.30 ***	0.34 ***	−0.15 **	0.05	0.35 ***	0.50 ***	0.32 **	1.00		
11. Masculinity traits	90.83	17.27	0.11 *	0.08	0.08	0.12 *	−0.17 **	0.23 ***	0.06	0.04	0.31 ***	0.24 ***	1.00	
12. Femininity traits	94.38	15.63	0.21 ***	−0.11	0.16 **	0.20 ***	−0.11 *	0.09	0.03	0.00	0.27 ***	0.20 ***	0.58 ***	1.00

Notes: (1) sex: 0 = women, 1 = men; marital status: 0 = not married, 1 = married; living arrangement: 1 = living with parent, 0 = not living with parent; rank: 0 = employees, 1 = managers. (2) * *p* < 0.05, ** *p* < 0.01, *** *p* < 0.001.

**Table 2:** Results of moderated regression on family-to-work conflict (FWC): masculinity traits.

Predictors	FWC				
	Model 1	Model 2	Model 3	Model 4	Model 5
**Control variables**					
Age	0.01	0.02	0.03	0.01	0.01
Marital Status	0.18	0.15	0.14	0.11	0.11
Living arrangement	0.00	0.02	0.02	0.01	0.01
Having children	0.08	0.11	0.11	0.14	0.13
Rank	−0.07	−0.10	−0.12 *	−0.14 *	−0.13 *
**Independent variable**					
Workload		0.17 **	0.19 **	0.24 ***	0.23 ***
**Moderators**					
Masculinity traits			−0.01	−0.03	−0.03
Sex			0.15 **	0.16 **	0.14 *
**2-way interactions**					
Workload × Masculinity traits				0.07	0.05
Workload × Sex				−0.15 *	−0.14 *
Sex × Masculinity traits				0.14 *	0.14 *
**3-way interaction**					
Workload × Sex × Masculinity traits					0.06
R^2^	0.07	0.09	0.12	0.15	0.15
∆R^2^	0.07 **	0.03 **	0.02 *	0.03 *	0.00
F	4.40 **	5.16 ***	4.87 ***	4.60 ***	4.31 ***
(df)	(5298)	(6297)	(8295)	(11,292)	(12,291)

Notes: (1) All coefficients are standardized beta coefficients. (2) Sex: 0 = women, 1 = men; Marital status: 0 = not married, 1 = married; Living arrangement: 1 = living with parent, 0 = not living with parent; Rank: 0 = employees, 1 = managers. (3) * *p* < 0.05, ** *p* < 0.01, *** *p* < 0.001.

**Table 3:** Results of moderated regression on FWC: femininity traits.

Predictors	FWC				
	Model 1	Model 2	Model 3	Model 4	Model 5
**Control variables**					
Age	0.01	0.02	0.04	0.03	0.03
Marital Status	0.21	0.17	0.15	0.15	0.13
Living arrangement	0.02	0.03	0.04	0.03	0.04
Having children	0.05	0.08	0.10	0.10	0.12
Rank	−0.08	−0.11	−0.13 *	−0.15 *	−0.15 **
**Independent variable**					
Workload		0.17 **	0.21 ***	0.25 ***	0.26 ***
**Moderators**					
Femininity traits			−0.06	−0.06	−0.07
Sex			0.16 **	0.17 **	0.14 *
**2-way interactions**					
Workload × Femininity traits				0.08	0.08
Workload × Sex				−0.12 *	−0.09
Sex × Femininity traits				0.12 *	0.11 *
**3-way interaction**					
Workload × Sex × Femininity traits					0.14 *
R^2^	0.06	0.09	0.12	0.15	0.17
∆R^2^	0.06 **	0.03 **	0.03 **	0.03 *	0.02 *
F	4.12 **	5.03 ***	5.07 ***	4.66 ***	4.81 ***
(df)	(5299)	(6298)	(8296)	(11,293)	(12,292)

Notes: (1) All coefficients are standardized beta coefficients. (2) Sex: 0 = women, 1 = men; Marital status: 0 = not married, 1 = married; Living arrangement: 1 = living with parent, 0 = not living with parent; Rank: 0 = employees, 1 = managers. (3) * *p* < 0.05, ** *p* < 0.01, *** *p* < 0.001.

**Table 4:** Results of moderated regression on work-to-family conflict (WFC): masculinity traits.

Predictors	WFC				
	Model 1	Model 2	Model 3	Model 4	Model 5
**Control variables**					
Age	−0.04	−0.07	−0.07	−0.07	−0.06
Marital Status	0.20	0.20	0.19	0.19	0.20
Living arrangement	−0.03	−0.02	−0.03	−0.03	−0.04
Having children	−0.01	−0.09	−0.09	−0.10	−0.11
Rank	0.08	0.08	0.09	0.09	0.09
**Independent variable**					
Family responsibility		0.33 ***	0.34 ***	0.35 ***	0.33 ***
**Moderators**					
Masculinity traits			−0.04	−0.04	−0.03
Sex			0.07	0.07	0.05
**2-way interactions**					
Family responsibility × Masculinity traits				−0.05	−0.06
Family responsibility × Sex				−0.03	−0.04
Sex × Masculinity traits				−0.04	−0.03
**3-way interaction**					
Family responsibility × Sex × Masculinity traits					0.09
R^2^	0.04	0.14	0.14	0.15	0.15
∆R^2^	0.04 *	0.09 ***	0.01	0.01	0.01
F	2.72 *	7.83 ***	6.12 ***	4.61 ***	4.44 ***
(df)	(5299)	(6298)	(8296)	(11,293)	(12,292)

Notes: (1) All coefficients are standardized beta coefficients. (2) Sex: 0 = women, 1 = men; Marital status: 0 = not married, 1 = married; Living arrangement: 1 = living with parent, 0 = not living with parent; Rank: 0 = employees, 1 = managers. (3) * *p* < 0.05, ** *p* < 0.01, *** *p* < 0.001.

**Table 5:** Results of moderated regression on WFC: femininity traits.

Predictors	WFC				
	Model 1	Model 2	Model 3	Model 4	Model 5
**Control variables**					
Age	−0.09	−0.12	−0.11	−0.11	−0.12
Marital Status	0.25 *	0.25 *	0.25 *	0.25 *	0.28 **
Living arrangement	−0.02	−0.02	−0.02	−0.02	−0.01
Having children	−0.02	−0.12	−0.12	−0.13	−0.13
Rank	0.09	0.09	0.09	0.09	0.09
**Independent variable**					
Family responsibility		0.34 ***	0.35 ***	0.35 ***	0.33 ***
**Moderators**					
Femininity traits			−0.03	−0.04	−0.03
Sex			0.06	0.06	0.04
**2-way interactions**					
Family responsibility × Femininity traits				0.02	0.01
Family responsibility × Sex				−0.05	−0.05
Sex × Femininity traits				−0.06	−0.05
**3-way interaction**					
Family responsibility × Sex × Femininity					0.14 *
R^2^	0.04	0.14	0.15	0.15	0.17
∆R^2^	0.04 **	0.10 **	0.01 **	0.01 *	0.02 *
F	2.67 **	8.29 ***	6.46 ***	4.90 ***	5.11 ***
(df)	(5,300)	(6,299)	(8,297)	(11,294)	(12,293)

Notes: (1) All coefficients are standardized beta coefficients. (2) Sex: 0 = women, 1 = men; Marital status: 0 = not married, 1 = married; Living arrangement: 1 = living with parent, 0 = not living with parent; Rank: 0 = employees, 1 = managers. (3) * *p* < 0.05, ** *p* < 0.01, *** *p* < 0.001.

## Data Availability

The data that support the findings of this study are available from the corresponding author, L. Lu, upon reasonable request.
